# Gromacs MetaDump: a tool for extracting GROMACS simulation metadata

**DOI:** 10.1186/s13321-025-01082-5

**Published:** 2025-10-23

**Authors:** Adrián Rošinec, Terézia Slanináková, Tomáš Pavlík, Róbert Randiak, Tomáš Svoboda, Tomáš Raček, Gabriela Bučeková, Ondřej Schindler, Matej Antol, Aleš Křenek, Karel Berka, Radka Svobodová

**Affiliations:** 1 CEITEC - Central European Institute of Technology, Masaryk University, Kamenice 753/5, 625 00 Brno, Czech Republic; 2 National Centre for Biomolecular Research, Faculty of Science, Masaryk University, Kamenice 753/5, 625 00 Brno, Czech Republic; 3 Institute of Computer Science, Masaryk University, Šumavská 525/33, 602 00 Brno, Czech Republic; 4 Department of Physical Chemistry, Faculty of Science, Palacky University, 17. listopadu 12, 779 00 Olomouc, Czech Republic

**Keywords:** GROMACS, Molecular dynamics, Metadata extraction, FAIR, GROMACS MetaDump, Simulation annotations

## Abstract

**Abstract:**

The volume of molecular dynamics (MD) simulation data shared via public repositories is rapidly increasing; however, fragmentation across multiple independent repositories, each employing distinct dataset identifiers and metadata schemas, hinders the efficient exploration and reuse of these data. In this study, we present GROMACS MetaDump, a tool for automatic annotation of output files from GROMACS MD simulations producing human- and machine-readable metadata leveraging the native GROMACS metadata (gmx dump) output. The tool takes the run input simulation file (.tpr) as the basis for the metadata output, optionally extending it with annotations from topology and structure files (.top, .gro). The tool is available as a web application (https://gmd.ceitec.cz/), API service, and a command-line utility. By automating the metadata extraction process, GROMACS MetaDump aims to simplify and standardise the extraction of rich, structured metadata from GROMACS MD simulations, making it easier to share, discover, and reuse simulation data within the research community.

**Scientific contribution:**

This work introduces GROMACS MetaDump, a software tool for the automatic extraction of metadata from molecular dynamics (MD) simulations performed with GROMACS. GROMACS MetaDump captures all extractable simulation parameters, such as software version, force field, water model, box geometry, temperature, etc., and returns them in a structured JSON or YAML file. As a result, GROMACS MetaDump supports the creation of unified metadata annotations of MD simulations, making datasets indexable and findable in line with the FAIR principles.

## Introduction

Molecular dynamics (MD) simulation is an indispensable computational approach for studying the structure, function, and dynamics of biological macromolecules, such as proteins, nucleic acids, and lipids. These simulations generate vast amounts of data that provide critical insights into molecular behaviour at atomic resolution.

Despite the significant scientific value of MD simulations, the data they produce are not accessible through a single community-accepted resource. Instead, they are being deposited into generalist repositories [1], such as Zenodo (https://zenodo.org), FigShare (https://figshare.com), or OSF (https://osf.io). More recently, various content-specific repositories [2–5] emerged, but none have yet gained widespread adoption. This fragmentation complicates the retrieval of past simulations and their efficient combination or comparison. The MD community is aware of this challenge and is addressing it through initiatives like the MDDB (https://mddbr.eu) [6], whose main goal is to develop a federation of data repositories for MD simulations that promotes data accessibility and aligns with the FAIR data principles [7].

However, an essential aspect of maximising the scientific potential of existing MD simulations is not only the availability of the data but also their practical usability [1]. In this context, high-quality metadata are recognised to significantly aid in the utility of MD data [1, 8], providing structured data context necessary for traditional research workflows as well as enabling advanced AI applications in MD [9, 10]. Their value is also increasingly reflected in formal academic requirements, with metadata now being specified as supplementary material in both publications and grant proposals, e.g. ERC [11] or Horizon Europe [12]. While a unified metadata schema for MD remains to be established, multiple works [1, 13, 14] converge on essential metadata annotations for MD data deposition. These consistently include simulation parameters (temperature, force field, etc.), composition of the system with identification of the simulated macromolecule, and simulation software with its version. Although the necessity of MD metadata is well-established, its practical implementation lags behind this recognition [15].

As a response, in this work we present a software tool, GROMACS MetaDump (https://gmd.ceitec.cz), which automatically extracts essential metadata annotations from MD simulations produced by GROMACS, one of the most broadly adopted software for MD simulations [1]), and converts them into machine-readable and processable format (JSON or YAML). The tool also allows for optional manual input of practical annotations that cannot be derived directly from the simulation. GROMACS MetaDump can be accessed through a web interface, an API, and a command-line utility. It is freely available at https://github.com/sb-ncbr/gromacs-metadump and requires no registration to use.

## GROMACS MetaDump

### Metadata model

GROMACS, a widely used molecular dynamics (MD) simulation package, does not define an explicit metadata model, but it inherently maintains detailed simulation information. Key input files such as .tpr, .top, and .gro collectively specify the simulation setup, including system topology, force fields, and parameters. Extracting metadata from these files provides a foundational metadata model for GROMACS-generated MD data, which we refer to as the GROMACS MetaDump Schema. Similar efforts to define standardised metadata models for MD simulations have been attempted in the past, such as the iBIOMES project [13] and the white paper on standards for data handling [14], but their adoption across the field remains limited. Consequently, various repositories and projects have implemented their own metadata schemas tailored to their specific needs. For instance, [3] include project information, topologies, and simulation parameters, while MDVerse emphasises temperature, simulation length, force field, and software metadata. Similarly, in [5] Banáš et al. extract metadata from AMBER simulation files, incorporating identifiers such as author names and publication dates but without proposing a standardised model. These examples highlight the fragmentation of metadata approaches in MD, reinforcing calls for unified metadata standards [1, 3]. In response to these challenges, our approach leverages GROMACS’ native information about simulation parameters, as they are most valuable, already defined, and describe the simulation experiment very well, as we use the input run file. We then enrich it with additional metadata such as software version, simulation object details, and author information. This aligns with ongoing standardisation efforts, ensures that critical simulation descriptors are consistently captured, and facilitates findability and interoperability across MD repositories.

**Fig. 1:**
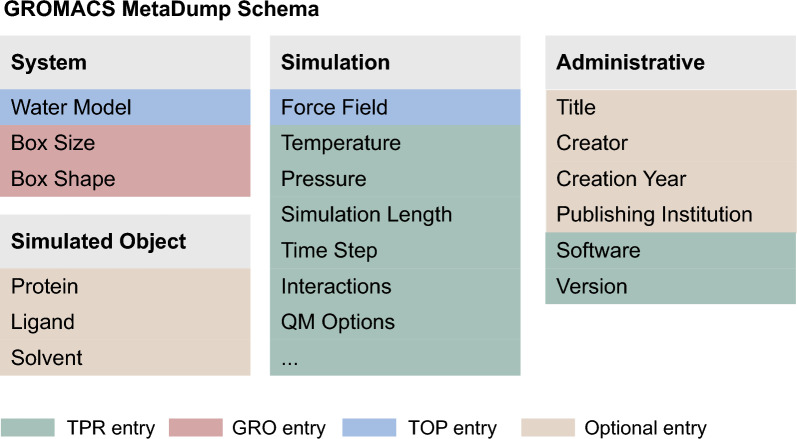
Metadata schema used in GROMACS MetaDump

Our proposed metadata schema for GROMACS MD data (see Fig. 1) consists of four logical parts. *System* annotations define the physical setup of the simulation—the water model and the box parameters. *Simulation* groups force field with all the parameters present in .tpr, such as temperature, pressure, or time step. *Simulated Object* captures the identifier of the simulated structure. For proteins, PDB or UniProt ID, for ligands PubChem, ChEMBL, DrugBank IDs, and for lipid complexes described in e.g. lipidmaps identifiers are expected. Finally, *Administrative* annotations include practical fields about the data creation, the GROMACS version used, and authors of the dataset. The schema is represented as JSON Schema and is available at following URL: https://gmd.ceitec.cz/schema/GROMACS-2025-1.schema.json. To provide a clearer understanding of how our metadata schema compares to previous efforts, Table S1 in Supplementary file 1 provides a detailed comparison of the GROMACS MetaDump Schema with existing metadata initiatives.

### Functionality

The core of the GROMACS MetaDump software centres on the automatic extraction of information from GROMACS files. The .tpr file is processed using our modification of GROMACS dump (gmx dump - https://manual.gromacs.org/current/onlinehelp/gmx-dump.html) tool, which is available at GitHub (https://github.com/sb-ncbr/gromacs). The annotations from .gro and .top files are parsed using regular expression pattern matching. In .top, GROMACS MetaDump parses the filenames of the included files containing the definition of water model and force field whereas in .gro, specific box properties are extracted. The extracted information is stored internally in JSON format and undergoes validation against a JSON Schema that implements the metadata model defined in Metadata Model.

The user manual is accessible through the web application and is also available at: https://github.com/sb-ncbr/gromacs-metadump/wiki/manual.

### Execution

The tool provides three distinct modes of access: a web application designed for ease of use, an API service for integration into automated workflows, and a command-line utility for programmatic access.

#### Input

The tool accepts GROMACS simulation files, specifically the required simulation run input file (extension .tpr), along with optional topology (.top) and structure (.gro) files. These files can be provided individually or bundled in a .zip or .tar (.gz) archive. Furthermore, the software supports the integration of optional annotations in the JSON format (see https://github.com/sb-ncbr/gromacs-metadump/wiki/Manual#optional-metadata for the expected structure) to enhance the resulting metadata.

#### Output

**Fig. 2:**
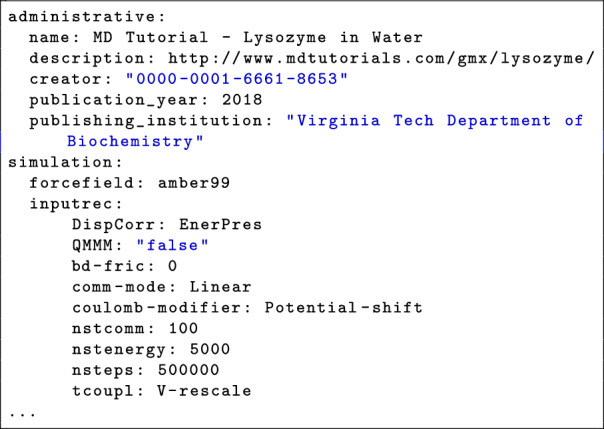
An example of output YAML file

The output is a JSON or YAML file containing simulation annotations. A shortened example of the generated YAML output is presented in Fig. 2, illustrating the format and content of the metadata annotations.

#### Web application

The web application interface has been developed to enable easy and user-friendly access to metadata generation from user input. Upon uploading the input file(s) (Section Input), the user can initiate the annotation process or optionally supply optional metadata. The website immediately redirects the user to an overview of uploaded files. The annotations can be downloaded in JSON or YAML format or viewed and edited directly in the application using the website’s metadata editor. The website stores a unique identifier for each annotation request in the user’s browser, allowing easy retrieval of previous requests as sessions. These sessions are retained for at least one month and are displayed on the front page for the user’s convenience. Finally, the website supports an optional user profile pre-fill feature, enabling quick insertion of optional metadata such as the creator’s name and publishing institution.

#### API

**Fig. 3:**
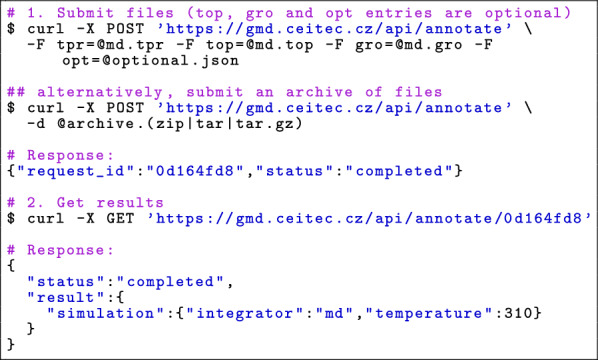
API workflow. The annotations can be found in the results attribute in the last response

The API interface facilitates automated metadata annotation for GROMACS simulation files. It supports individual file uploads and bulk archive submissions, making it suitable for integration into automated workflows. Figure 3 shows an example of the API communication to retrieve the annotations. The process consists of two steps. First, a POST request with files to upload is executed to trigger the annotation extraction, then a GET request with a unique identifier is performed to obtain the annotations. The complete API documentation is available in https://github.com/sb-ncbr/gromacs-metadump/wiki/Manual#api-usage.

#### Command-line utility

**Fig. 4:**
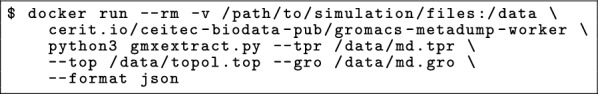
Running GROMACS MetaDump as a command-line utility

The final option for executing GROMACS MetaDump is via a command-line utility. This option eliminates the necessity for transmitting MD data over the internet, thereby ensuring on-premise functionality. We provide the command-line as a script and as a Docker container, since we are employing a modified version of the GROMACS dump tool. The user is required to mount the data into the running container, execute the script, and the metadata annotations are printed to the terminal, see Fig. 4.

### Workflow

The GROMACS MetaDump extraction process starts with the submission of input files described in Sect. Input. If an archive is submitted, its contents are extracted before processing. Next, GROMACS MetaDump extracts annotations from the available sources. For the .tpr file, it uses uses gmx dump, for .top and .gro (if provided) it applies regular expression (Regex) matching. These extracted values are assembled into a preliminary JSON annotation.

**Fig. 5:**
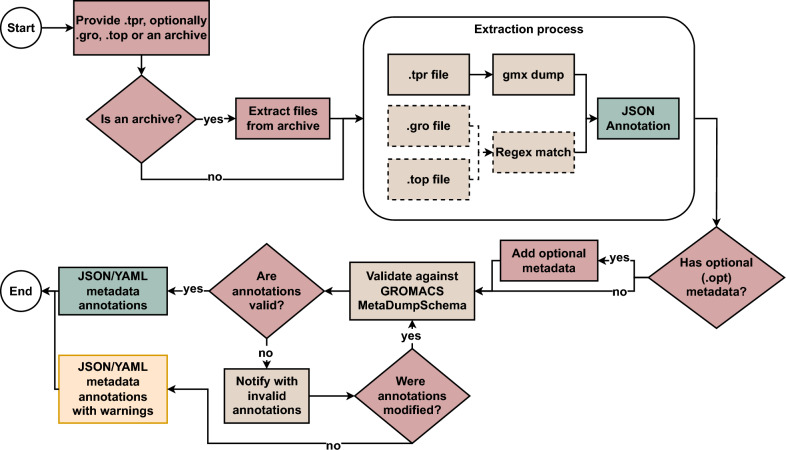
The GROMACS MetaDump metadata extraction workflow

If optional metadata entries are included, they are merged into the JSON output. The resulting annotations are then validated against GROMACS MetaDump Schema (see Fig. 1) to ensure structural and semantic correctness. Upon successful validation, the final metadata is made available in both JSON and YAML formats. In the event of a validation error, the user is prompted to correct the identified issues. This workflow is illustrated in Fig. 5.

### Implementation

The web application’s graphical interface is built with JavaScript using React.js, with GUI components from Material UI. It integrates a library for displaying metadata annotations in JSONForms, which automatically generates input fields based on a defined JSON Schema. The backend API service handles requests and runs GROMACS MetaDump based on the GROMACS 2024.3 version. The API is built with Python 3.12 and uses Flask for routing and a WSGI server to process HTTP requests. The backend manages a queue of incoming requests and calls the processing functions and parsers.

### Limitations

The current version of the JSON schema for describing datasets is focused on datasets created by GROMACS. That is, the schema uses attribute names based on the GROMACS parameters. Our metadata annotation extraction implementation is limited to GROMACS-generated datasets. The latest version of GROMACS MetaDump built on GROMACS 2025.1 is working for all current datasets created after 2015. See the Table S2 in the Supplementary material 2 showing the versions of GROMACS used to generate running files (.tpr), which could be parsed by the GROMACS MetaDump.

GROMACS MetaDump currently supports a subset of all GROMACS simulation file types. This limitation affects the annotation of water model and force field parameters. GROMACS MetaDump infers these annotations from the filenames of .itp files included in the analysed .top file, which may not always accurately reflect their actual contents–especially in cases involving custom or modified files. We are also not capable of obtaining force-fields parameters (e.g. atom charges, vwd parameters, etc.) applied to the experiment in the setup phase—only resulting parameters GROMACS is capable to export from files such as .tpr.

## Results and discussion

We evaluated the application through user testing to ensure its stability and reliability across 4364 GROMACS simulations obtained from generalist repositories (Zenodo, FigShare, OSF) as of April 15, 2025. These simulations span GROMACS versions from 4.5 to 2025. Additionally, the web application includes three example cases that demonstrate the use of GROMACS MetaDump without needing to upload any input files. First, *Lysozyme in Water* from the GROMACS Tutorial (http://www.mdtutorials.com/gmx/lysozyme/). Next, *Helicobacter pylori TonB-CTD* from [16] discussed in detail in Sect. Use-case A: annotation of helicobacter pylori TonB-CTD simulation. Finally, *Cryo-EM structure of human KATP bound to ATP and ADP in quatrefoil form* published in [17].

In the following sections, we present two use cases that demonstrate how to leverage and interpret GROMACS MetaDump annotations.

### Use-case A: annotation of helicobacter pylori TonB-CTD simulation

TonB protein is responsible for energy transduction involved in transporting ferric iron complexes and vitamin B12 across the outer membrane of Gram-negative bacteria. This process involves interactions between the C-terminal domain of the TonB protein and the TonB box of TonB-dependent transporters (TBDTs) [18, 19].

Ollila et al. [16] describe nine simulations, focusing on the C-terminal domain of the TonB proteins: HpTonB-92 (PDB ID: *5lw8*) and PaTonB-96 (PDB ID: *6fip*). The paper also provides a link to the simulation files deposited to Zenodo. For each experiment, we downloaded the relevant simulation files (.tpr, .gro, .top) and used them as input to GROMACS MetaDump. The tool generated metadata annotations with 86 distinct fields, providing significantly more detailed information than the description presented in [16]. With these, we were able to reproduce the metadata presented in the paper and found several subtle but important differences (see Table 1). While the core simulation setup parameters (force field, temperature, pressure, etc.) were consistent, we identified variations in two categories:

1. **Water Model**: Four simulations from [16] reportedly use the tip4p water model, while GROMACS MetaDump identified the model as tip4pew within the shared simulation data. While subtle, the distinction between these can influence simulation outcomes, as tip4pew is specifically reparameterized for use with Ewald summation methods like PME, including changes to Lennard–Jones parameters and charge placement. Without clear labelling, such distinctions can easily be missed, leading to incorrect assumptions about the system’s physical behaviour or force field fidelity. Similarly, in two simulations from [16], Ollila et al. report using OPC4 water model while GROMACS MetaDump identified the model as OPC. This again represents a subtle but significant difference. While both are 4-point water models, OPC4 is a refined version of OPC with modified charge distributions, adjusted geometry parameters, and optimised performance characteristics for specific simulation conditions. This initial discrepancy arose because the water model was included via a custom opc.itp file, which was referenced in the .top file. Since GROMACS MetaDump relies on parsing the include directive and does not analyse the contents of .itp files directly, it interpreted the model based solely on the filename. Manual inspection of the opc.itp file confirmed that it actually corresponds to OPC4, not OPC. Thus, while the simulation did indeed use OPC4 as reported, this case underscores the importance of clear and consistent file naming when sharing simulation data, to avoid ambiguity and misinterpretation.
2. **Simulation time**: While not explicitly present in the GROMACS MetaDump metadata, simulation time can be derived from the time step (dt) and number of steps (nsteps) ($50,000,000 \times 0.002~\text {ps} = 100,000~\text {ps} = 100~\text {ns}$). This calculated value differs from both the total simulation time and analysed simulation time reported in Table 1 (columns 4, 5) of [16]. The presence of a “continuation”: “true” field in the metadata suggests these simulation files represent a continuation of a previous simulation. Extraction of the complete temporal information would require the analysis of both the .tpr and .xtc files.

**Table 1:** Comparison of simulation parameters between reported values in [16] and those extracted from GROMACS MetaDump. The identified discrepancies are shown in bold

Parameter name	Annotation field in GROMACS MetaDump	Found with GROMACS MetaDump	Reported in [16]
Software version	software_information	GROMACS 5.1.4	GROMACS 5
Force field	forcefield	amber99sb-ildn	Amber ff99SB-ILDN
Water model	water_model	tip3p, **tip4pew**, **opc**	tip3p, **tip4p**, **OPC4**
Temperature	ensemble-temperature	298, 303, 310 K	298, 303, 310 K
Pressure coupling	pcoupl	Parrinello-Rahman	Parrinello-Rahman
Pressure coupling type	pcoupltype	Isotropic	Isotropic
Time step	dt	0.002 ps	2 fs
Number of steps	nsteps	50000000	–
Simulation time	–	**100 ns**	**200–1190 ns**

### Use-case B: metadata overview on simulation datasets from generalist repositories

**Fig. 6:**
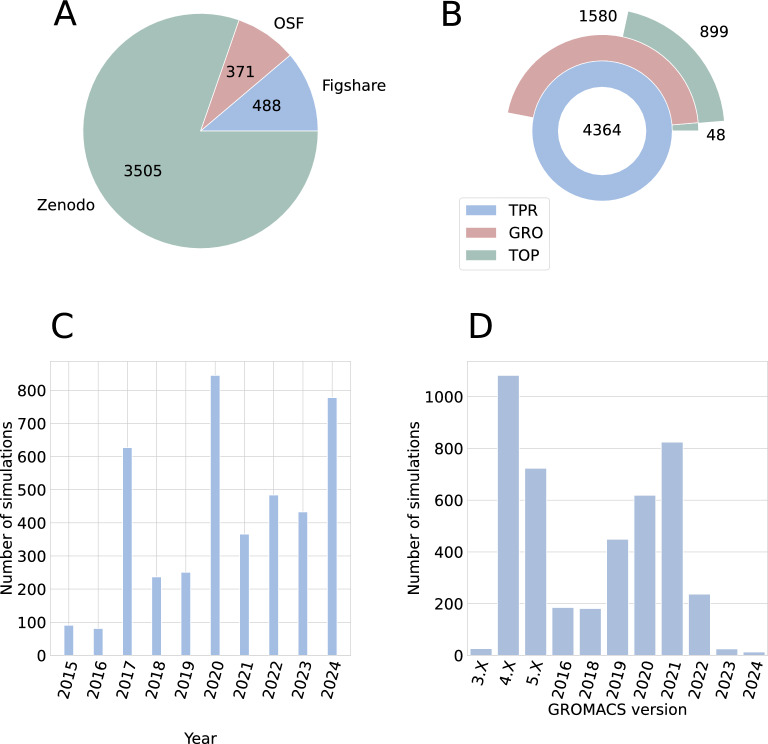
Results of basic analyses of selected metadata from publicly available GROMACS MD simulations (Part 1): **A** Distribution of MD simulation data in generalist data repositories. **B** File structure of simulation uploads. **C** Number of GROMACS simulations published per year. **D** GROMACS version (series) used in deposited simulations.Detailed list of all GROMACS versions found are available in Table S2 included in Supplementary file 2

**Fig. 7:**
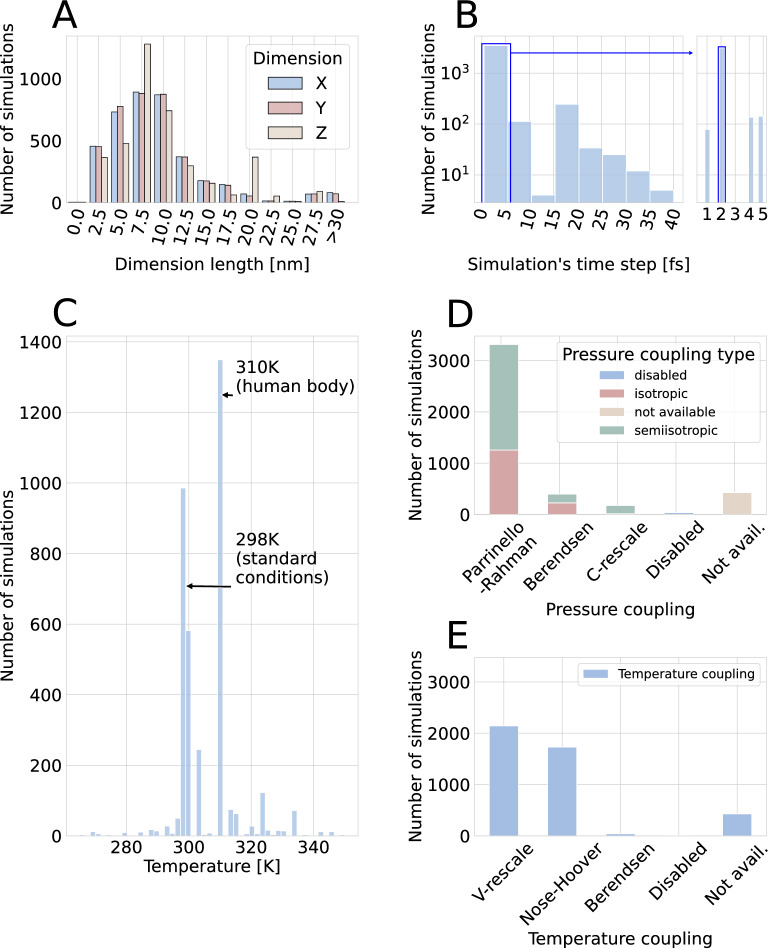
Results of basic analyses of selected metadata from publicly available GROMACS MD simulations (Part 2): **A** Distribution of simulation box dimensions across X, Y, and Z axes. **B** Distribution of simulation time steps. **C** Temperature distribution of simulations. **D** Distribution of pressure coupling algorithms. **E** Distribution of temperature coupling algorithms

In this use case, we demonstrate the usefulness of accumulating machine-readable metadata to gain a comprehensive overview of many simulations. We performed several basic analyses studying the broad simulation trends shown in the metadata obtained by GROMACS MetaDump from 4364 MD simulation files. These represent all simulations containing a .tpr file available in major generalist data repositories as of April 15, 2025. The results of these analyses are presented in Figs. 6 and 7.

The analysis of repository usage reveals a clear preference for Zenodo, which hosts over 80% of the datasets [Fig. 6A]. The distribution of file types reflects typical GROMACS workflows [Fig. 6B], where TPR files are essential as they contain complete simulation parameters, while structure files (GRO) and topology files (TOP) are sometimes omitted from public deposits. The temporal analysis demonstrates growing adoption of data sharing practices in the molecular dynamics community, with a significant surge during 2020 [Fig. 6C], possibly influenced by increased remote collaboration needs during the global pandemic. The version distribution [Fig. 6D] suggests that many research groups maintain stable GROMACS installations, with version 4.6 still widely used.

The physical parameters of the simulations align with common biomolecular research practices. The predominant box dimensions of 5–15 nm [Fig. 7A] are typical for protein-water systems, providing sufficient space to prevent self-interaction across periodic boundaries. The near-universal adoption of 2 fs time steps [Fig. 7B] represents a standard compromise between simulation accuracy and computational efficiency, enabled by constraint algorithms for hydrogen-containing bonds. Temperature choices [Fig. 7C] reflect the field’s focus on physiologically relevant conditions, with simulations clustered around room temperature (298K) and human body temperature (310K). The most widely used pressure coupling algorithm is Parrinello-Rahman [Fig. 7D]. V-rescale and Nose-Hoover approaches are the most frequently used temperature coupling algorithms [Fig. 7E].

## Conclusions

In this work, we introduced GROMACS MetaDump, a comprehensive tool for automated metadata extraction and annotation of GROMACS molecular dynamics simulations. The tool is freely available as a web application, through a public API and as a command-line utility. GROMACS MetaDump processes GROMACS input files, extracting and organizing simulation parameters into structured metadata annotations. These annotations adhere to the GROMACS MetaDump Schema, which we developed based on GROMACS’s internal parameter cataloging system. We demonstrated the utility of GROMACS MetaDump through two comprehensive use cases. First, we used GROMACS MetaDump to extract metadata from the deposited simulation files in [16] and contrasted them with the simulation properties reported in the paper. This analysis revealed subtle discrepancies in temporal parameters and water model implementations. Second, we conducted a systematic analysis of over 4000 GROMACS simulations, highlighting key trends in simulation practices, such as deposition patterns over time, GROMACS version adoption, and file sharing practices.

The tool itself can help the user to navigate through the current “dark matter” of molecular dynamics data by giving a standardised, quick insight to the problem “I’ve found this simulation in the MD ‘dark matter’ data on Zenodo, is it relevant for me to compare with my results?”. Moreover, we believe that machine-readable metadata are essential for making molecular dynamics simulations more accessible and reusable. As the volume of published MD data continues to grow, GROMACS MetaDump, with its automated approach to metadata augmentation, can contribute to building the infrastructure for sharing and analysing simulation data at scale. Our platform-independent tool may support initiatives like the European MDDB project by automating metadata gathering and promoting standardised metadata practices. As such, this work is the next step on the way towards truly FAIR molecular dynamics data repositories.

## Additional file

**Additional file 1.**

**Additional file 2.**

## Data Availability

**Project name:** GROMACS MetaDump **Project home page:** https://gmd.ceitec.cz/ **Operating system(s):** Platform independent **Programming language:** Python **Other requirements:** None for Web and API access; GROMACS and Python 3.8+ or Docker for CLI access **License:** BSD 3-Clause License
