# Supplementary material for: Gromacs MetaDump: a tool for extracting GROMACS simulation metadata
Source: J Cheminform. 2025 Oct 23;17:160. doi: 10.1186/s13321-025-01082-5 (PMC12548288; doi:10.1186/s13321-025-01082-5)
Supplement: Supplementary file 1 — Additional file 1. [file 13321_2025_1082_MOESM1_ESM.pdf]

Table S1: Comparison of the attributes present in the proposed GROMACS MetaDump schema with four previously proposed metadata schemas for MD.

	**GROMACS MetaDump**	**iBIOMES**	**ScalaLife**	**COVID-19**	**IDA**
**Administrative Parameters**					
Authors/creators	✓	✓	✓	✓	✓
DOI/persistent identifiers	✓	×	×	×	✓
Publishing institution	✓	×	×	×	×
Year/Date of publishing	✓	×	×	×	✓
Software used	✓	✓	✓	✓	✓
Program version	✓	×	✓	✓	✓
**System Parameters**					
Water model	✓	×	✓	✓	×
Box size/shape	✓	×	✓	×	✓
Molecule counts	✓	✓	✓	×	✓
**Simulation Parameters**					
Force field	✓	✓	✓	✓	✓
Simulation length	✓	✓	✓	✓	✓
Time step	✓	×	✓	×	✓
Statistical ensemble	✓	×	✓	×	✓
Temperature/pressure	✓	×	✓	✓	✓
Thermostat/barostat	✓	✓	×	×	✓
Electrostatics interactions	✓	✓	✓	×	✓
Constraint algorithm	✓	×	✓	×	×
van der Waals settings	✓	×	×	×	✓
Neighbor list parameters	✓	×	×	×	×
**Simulated Object Parameters**					
Molecule type	✓	✓	✓	✓	✓
Solvent	✓	✓	✓	×	✓
Molecule identification	✓	×	✓	✓	×
