# Supplementary material for: Gromacs MetaDump: a tool for extracting GROMACS simulation metadata
Source: J Cheminform. 2025 Oct 23;17:160. doi: 10.1186/s13321-025-01082-5 (PMC12548288; doi:10.1186/s13321-025-01082-5)
Supplement: Supplementary file 2 — Additional file 2. [file 13321_2025_1082_MOESM2_ESM.pdf]

Table S2: Count of GROMACS simulations per version of the software, grouped by major version series. Data collected from Zenodo, FigShare, and OSF repositories in March 2025.

Series	Version	Count
2024 series	2024.2	4
	2024.1	5
	2024	4
2023 series	2023.1-EasyBuild_4.8.0	3
	2023.1	21
	2023	1
2022 series	2022.6	12
	2022.6-plumed_2.9.0	8
	2022.5-plumed_2.8.2	44
	2022.5-Debian_2022.5_2	3
	2022.5	3
	2022.4	15
	2022.4-dev	8
	2022.3-spack	6
	2022.3	29
	2022.2	3
	2022-beta1	6
	2022	100
2021 series	2021.7	4
	2021.6-Colvars-2022-10-24-dev	3
	2021.5-plumed-2.7.6	4
	2021.5-EasyBuild-4.7.1-PLUMED-2.8.0	48
	2021.5-spack	122
	2021.5	74
	2021.5-EasyBuild-4.7.0-PLUMED-2.8.0	3
	2021.4-plumed-2.8.0	53
	2021.4	5
	2021.4-EasyBuild-4.5.0	8
	2021.4-plumed-2.9.0-dev	27
	2021.4-plumed-2.7.3	24
	2021.4-Ubuntu-2021.4-2	1
	2021.3	129
	2021.3-MODIFIED	24
	2021.2-MODIFIED	44
	2021.2-plumed-2.7.6	44
	2021.2-plumed-2.8.2	8
	2021.2	51
	2021.1	104
	2021-UNCHECKED	1
	2021	43

Series	Version	Count
2020 series	2020.6-Colvars-2021-12-20-dev	2
	2020.6	11
	2020.5-MODIFIED	3
	2020.5	179
	2020.4-MODIFIED	1
	2020.4	66
	2020.3	16
	2020.2	7
	2020.1	118
	2020.1-Ubuntu-2020.1-1	195
	2020.1-MODIFIED	6
	2020-UNCHECKED	2
	2020	13
2019 series	2019.6	50
	2019.5	22
	2019.4	166
	2019.3	14
	2019.2	189
	2019.1	6
	2019	2
2018 series	2018.8	14
	2018.7	41
	2018.6	34
	2018.6-dev-20190220-75dc045	1
	2018.5	3
	2018.4	8
	2018.3	51
	2018.2	8
	2018.1	4
	2018	17
2016 series	2016.6	1
	2016.5	23
	2016.4	48
	2016.3	85
	2016.2	6
	2016.1	5
	2016	17

Series	Version	Count
5.X series	5.1.5	23
	5.1.4	220
	5.1.3-dev-20160627-f16daab	7
	5.1.2	70
	5.1.1	2
	5.1	27
	5.1-beta1	1
	5.1-dev-20150223-461b58f	1
	5.0.7	153
	5.0.6	65
	5.0.6-dev-20150529-78553ee	37
	5.0.5	11
	5.0.4	106
4.X series	4.6.8-dev-20141007-f2007ba	7
	4.6.7	177
	4.6.7-dev-20140707-0292846	15
	4.6.6-dev-20140221-6b6bb42	6
	4.6.6-dev-20140109-a052b75	4
	4.6.6	45
	4.6.5	456
	4.6.3-dev-20130628-9cd3bcc	23
	4.6.3	81
	4.6.2	3
	4.6.2-dev-20130329-bf4da54	1
	4.6	106
	4.5.7	7
	4.5.6-dev-20130119-a08a57c	1
	4.5.6	14
	4.5.6-dev-20130307-90abdcf	3
	4.5.4	10
	4.5.3	11
	4.5.1	9
	4.5.5	60
	4.5.5-LS	1
	4.5.5-dev-20120703-fc032f9-dirty	4
	4.0.7	20
	4.0.5	6
	4.0.2_localpressure	6
	4.0.2	6
3.X series	3.3.3	13
	3.1.4	12
	3.0.3	1
